# Destroy to Rebuild: The Connection Between Bone Tissue Remodeling and Matrix Metalloproteinases

**DOI:** 10.3389/fphys.2020.00047

**Published:** 2020-02-05

**Authors:** Eugenio Hardy, Carlos Fernandez-Patron

**Affiliations:** ^1^Center of Molecular Immunology, Havana, Cuba; ^2^Department of Biochemistry, Faculty of Medicine & Dentistry, University of Alberta, Edmonton, AB, Canada

**Keywords:** bone, remodeling, metabolism, matrix metalloproteinase, deficiency, underactivity

## Abstract

Bone is a dynamic organ that undergoes constant remodeling, an energetically costly process by which old bone is replaced and localized bone defects are repaired to renew the skeleton over time, thereby maintaining skeletal health. This review provides a general overview of bone’s main players (bone lining cells, osteocytes, osteoclasts, reversal cells, and osteoblasts) that participate in bone remodeling. Placing emphasis on the family of extracellular matrix metalloproteinases (MMPs), we describe how: (i) Convergence of multiple protease families (including MMPs and cysteine proteinases) ensures complexity and robustness of the bone remodeling process, (ii) Enzymatic activity of MMPs affects bone physiology at the molecular and cellular levels and (iii) Either overexpression or deficiency/insufficiency of individual MMPs impairs healthy bone remodeling and systemic metabolism. Today, it is generally accepted that proteolytic activity is required for the degradation of bone tissue in osteoarthritis and osteoporosis. However, it is increasingly evident that inactivating mutations in MMP genes can also lead to bone pathology including osteolysis and metabolic abnormalities such as delayed growth. We argue that there remains a need to rethink the role played by proteases in bone physiology and pathology.

## Introduction

Bone is a hard, dense, rigid form of highly specialized connective tissue making up the skeleton of vertebrates. Bone protects internal organs, supports body structures, and aids in locomotion (Maffioli and Derosa, 2015). In addition, bone provides an environment for hematopoiesis (i.e., formation and development of blood cells) in the bone marrow, and acts as a homeostatic reservoir of calcium, phosphorus, insulin-like growth factors, transforming growth factor-β, and cytokines. Bone buffers the blood against drastic pH changes, thus detoxifying the circulation from heavy metals (Rauner et al., 2012). Bone develops by intramembranous ossification (e.g., bone of the clavicle, some skull bones), endochondral ossification (e.g., the appendicular and axial skeleton) or pseudo-metamorphic ossification (Rauner et al., 2012).

Bone remodeling is a complex process involving the sequential resorption of bone tissue and deposition of new bone at the same site (Kerschan-Schindl and Ebenbichler, 2012). Together with bone structure, geometry, size, and density, remodeling determines bone’s overall mechanical properties (e.g., the strength) (Mosekilde et al., 1993; Jiang et al., 1997; Ikeda et al., 2003; Shahnazari et al., 2009) as well as enables the repair of damaged bone and the adaption of bone to changing biomechanical forces (Kerschan-Schindl and Ebenbichler, 2012).

We review here the prevailing view of the bone remodeling process with an emphasis on well-accepted and newly emerging roles played by matrix metalloproteinases (MMPs) and cysteine proteinases in this process. Finally, we review the increasing number of instances in which inactivating mutations in MMP genes are found to lead to bone pathology including osteolysis and metabolic abnormalities such as delayed growth.

## General Overview on the Cycle of Bone Remodeling

The bone remodeling process consists of four distinct consecutive phases spanning over 3–6 months (Datta et al., 2008).

The first phase of bone remodeling is known as the ‘activation phase’ and can be triggered by mechanical and nutritional stress on the bone as well as by hormones (e.g., parathyroid hormone, estrogen) (Parra-Torres et al., 2013). As described in Table 1, terminally differentiated osteocyte cell is a key player in the activation phase (Rauner et al., 2012; Parra-Torres et al., 2013).

**TABLE 1:** Osteocytes and the activation phase of bone remodeling.

Origin of osteocytes	Differentiation stages: (i) From mature osteoblasts to preosteocyte type I with dendritic projections formation; (ii) from preosteocyte type I to preosteocyte type II with cytoskeleton rearrangement; (iii) from preosteocyte type II to preosteocyte type III (mature osteocyte trapped within the mineralized bone matrix) with canaliculae formation (Hirao et al., 2007; Paiva and Granjeiro, 2017).
Important factors involved in osteocytogenesis	(i) Pre-osteoblasts (Stro1, CD29, CD105, CD166); (ii) Osteoblast (Cbfa1 and osterix for differentiation, alkaline phosphase and collagen for the production of osteoid, osteocalcin, casein kinase II); (iii) Osteoid osteocyte (Phex and MEPE for regulation of biomineralization and mineral metabolism, E11/gp38 and MMP-14 for dendrite/canaliculi formation, destrin for cytoskeleton rearrangement); (iv) Mineralizing osteocyte (DMP1 for regulation of biomineralization and mineral metabolism, CapG for cytoskeleton regulation); (v) Mature osteocyte (sclerostin, FGF23 for regulation of renal phosphate excretion, ORP150 for preserving viability in a hypoxic environment) (Bonewald, 2011). Other factors include TGF-β (D’Angelo et al., 2001; Karsdal et al., 2002), MMP-2/MMP-13/MMP-14 proteolytic axis (Barthelemi et al., 2012), Cx43, Dkk-1, Fetuin A, RANKL, MCS-F, and osteoprotegerin (Chen et al., 2018).
Key signaling events involved in osteocytogenesis	Osteocalcin, ALP, and other genes specific for osteoblast differentiation gradually downregulate (Paiva and Granjeiro, 2017). At the same time, different genes specific for osteocyte differentiation upregulate (such as CD44 [Hughes et al., 1994], E11/gp38 [Zhang et al., 2006], Phex [Ruchon et al., 2000; Westbroek et al., 2002], Fimbrin [Tanaka-Kamioka et al., 1998], MEPE [Rowe et al., 2004], DMP1 [Feng et al., 2006; Toyosawa et al., 2012], sclerostin [Poole et al., 2005; Balemans et al., 2008], ORP150 [Gao et al., 2010], and FGF23 [Liu et al., 2006]). Transcription factors involved in the process of osteoblast/osteocyte transition are ATF-4, whose expression is regulated by JNK, and members of the AP-1 group (Matsuguchi et al., 2009).
Role of osteocytes	(i) Maintain physical connections with each other, and also other players (osteoclasts, osteoblasts) of the bone remodeling cycle through a widespread network of tiny channels called canaliculi (Civitelli, 2008). (ii) May remodel the perilacunar matrix (e.g., during lactation) by expressing cathepsin K and acid phosphatase. (iii) Regulate bone remodeling by expressing M-CSF and RANKL (stimulate osteoclast formation and activity) as well as NO and OPG (inhibit osteoclast formation and activity). Also, osteocytes control bone formation by secreting activators (e.g., NO, ATP, PEG2) and inhibitors (e.g., sFRP1, DKK1, sclerostin) of the Wnt signaling pathway. (iv) Source of factors (e.g., sclerostin) and regulators (e.g., FGF-23, DMP-1, Phex, MEPE) of phosphate metabolism. (v) Manage the bone’s reservoir of calcium. (vi) Function as mechanosensory cells (Bonewald, 2011; Dallas et al., 2013; Bellido, 2014).
Molecular mechanism that underlies the function of osteocytes as mechanosensory cells	Osteocytes are good mechanosensors (i.e., they detect changes of mechanical stimuli) in bone tissue which serve to sense and respond to alterations produced when a bone is mechanically loaded. Such alterations may be physical deformation of the bone matrix, fluid flow shear stress generated by variations in canalicular fluid flow and electrical streaming potentials (Bonewald and Mundy, 1990; Mundy, 1993; Manolagas, 2000; Miyauchi et al., 2000; Bonewald and Johnson, 2008; Datta et al., 2008; Parra-Torres et al., 2013; Takemura et al., 2019). Mechanical strain signal is converted into a cellular response (i.e., biochemical signals) with the participation of membrane proteins (such as CD44, connexins, integrins, and ion channels) and downstream mediators of intracellular signaling (such as guanine regulatory proteins, mitogen activated protein kinase, cyclic adenosine monophosphate, inositol triphosphate, and intracellular calcium) (Rawlinson et al., 1996; Burger and Klein-Nulend, 1999; Mikuni-Takagaki, 1999; Miyauchi et al., 2000; Gu et al., 2001; Alford et al., 2003; Kapur et al., 2003; Plotkin et al., 2005; Rubin et al., 2006; Miyauchi et al., 2006). On the other hand, bone remodeling is also controlled by upregulation of RANKL and sclerostin in response to a decrease in mechanical signals (Parra-Torres et al., 2013). The precise signaling biochemical pathways (e.g., Wnt/β-catenin) and regulatory mechanisms that may mediate adaptive responses activated by mechanical loading and unloading in bone remain to be completely delineated (Dallas et al., 2013; Parra-Torres et al., 2013).
Other consequences of osteocyte activities on bone remodeling	Retraction of the bone lining cells (elongated mature osteoblasts) on the endosteal surface (which is a thin layer of cell-rich connective tissue), and also digestion of the underlying collagenous membrane by collagenases (Murray et al., 1995; Karsdal et al., 2001; Datta et al., 2008; Kerschan-Schindl and Ebenbichler, 2012).

*MMP, matrix metalloproteinase; TGF, transforming growth factor; RANKL, receptor activator of nuclear factor kappa B ligand; MAPK, mitogen-activated protein kinase; TIMP, tissue inhibitor of metalloproteinase; ALP, alkaline phosphatase; Phex, phosphate-regulating endopeptidase homolog X-linked; MEPE, matrix extracellular phosphoglycoprotein; Cx, connexin; Dkk, Dickkopf WNT signaling pathway inhibitor; Phex, phosphate-regulating endopeptidase homolog X-linked; DMP, dentin matrix acidic phosphoprotein precursor; M-CSF, Macrophage colony-stimulating factor; ORP, oxygen regulated protein; FGF, fibroblast growth factor; ATF, activating transcription factor; JNK, c-Jun N-terminal kinase; AP, activator protein; CD29, integrin beta-1; CD105, endoglin; CD166, activated leukocyte cell adhesion molecule; CapG, capping actin protein, gelsolin like; ORP150, 150-kDa oxygen-regulated protein; OPG; osteoprotegerin, ATP, adenosine triphosphate; NO, nitric oxide; sFRP1, secreted frizzled-related protein 1; DKK1, Dickkopf WNT signaling pathway inhibitor 1; PEG2, prostaglandin E2; Wnt, Wingless-type MMTV integration site family.*

The second phase lasts 8–10 days (Teitelbaum, 2007) and is called the ‘bone resorption phase’ – a process by which large multinucleated osteoclast cells break down old bone organic matrix impregnated with minerals (e.g., calcium phosphate nanocrystals), as described in Table 2.

**TABLE 2:** Osteoclasts and the bone resorption phase.

Origin of osteoclasts	Differentiation stages: Hematopoietic stem cell precursors differentiate into monocyte and macrophage, and then they fuse into end-differentiated multinucleated (bone resorbing) cells (Tanaka et al., 1993; Quinn et al., 1998; Roodman, 1999; Udagawa et al., 1999; Holmbeck and Szabov, 2006; Bar-Shavit, 2007; Bruzzaniti and Baron, 2006). Osteocyte apoptosis is thought to contribute to the recruitment of osteoclast precursors by diminishing the secretion of osteocyte-derived factors (e.g., TGF-β) that have inhibitory effect on osteoclast formation (Heino et al., 2002; Aguirre et al., 2006).
Main factors involved in osteoclastogenesis	Osteoblasts, osteocytes, RANKL, M-CSF, OPG, TNF, ILs, mineralized bone particles containing osteocalcin, DC-STAMP, OC-STAMP (Tanaka et al., 1993; Roach, 1994; Wiebe et al., 1996; Kotake et al., 1999; Udagawa et al., 1999; Marie, 2003; Miyamoto, 2006; Kim et al., 2011; Hienz et al., 2015; Plotkin and Bruzzaniti, 2019).
Key signaling events involved in osteoclastogenesis	After the induction of PU.1, the stem cell precursor is determined to the osteoclastic lineage (Tondravi et al., 1997). Then, cell proliferation is induced following expression and activation of *c-fms* by the precursor. RANK is subsequently expressed and activated by RANKL, after which RANK interacts with the TRAF family members (e.g., TRAF2, TRAF6) and lead to downstream activation of MAP kinases and NF-kβ. This process is aided by co-signaling from other receptors (such as TREM2, OSCAR, DAP 12, and FcRγ) (Koga et al., 2004; Mocsai et al., 2004). The interaction between immunoreceptors (e.g., TREM2, OSCAR) and FcRγ/FcRc adapters activates Syk kinases, leading to PLCγ activation. Ca(II), which is mobilized from the intracellular stores, activates calcineurin, resulting in dephosphorylation of NFATc1. Moreover, the activation of calcineurin involves the activation of phospholipase-Cγ and Tec kinases (Mocsai et al., 2004; Faccio et al., 2005; Wada et al., 2005). In general, most signaling pathways (MAPKs, NF-κB, AP-1, Ca(II), Src/PI3K/AKt) which are activated in the osteoclast converge to induce the activity of NFATc1 (Gori et al., 2000; Ishida et al., 2002; Takayanagi et al., 2002; Matsuo et al., 2004; Paiva and Granjeiro, 2017; Plotkin and Bruzzaniti, 2019; Zheng et al., 2019). Upon translocation to the nucleus, NFATc1 acts together with c-fos to promote the expression of key osteoclast genes. Some of the osteoclast differentiation genes to which NFATc1 binds directly are OSCAR (Kim Y. et al., 2005), cathepsin K (Matsumoto et al., 2004), calcitonin receptor (Matsuo et al., 2004), integrin β3 (Crotti et al., 2006, 2008), MMP-9 (Sundaram et al., 2007), and TRAP (Matsuo et al., 2004; Paiva and Granjeiro, 2017). Of note, another factor which controls NFATc1 is OPG, which functions as a decoy receptor for RANKL, thus inhibiting the differentiation of osteoclasts (Lacey et al., 1998). Osteoclastogenesis is regulated by the RANKL/OPG balance. Opposing effects on RANK during osteoclast differentiation is exerted by LGR4 which signals through G-protein or Wnt signaling pathways (Luo et al., 2016). Cytokines which inhibit RANK signaling on osteoclasts are IL-10, IFNs (α, β), and GM-CSF.
Mechanisms that underlie the action of osteoclasts	During initiation of the resorption phase, the mature osteoclasts (1-2% of bone cells) attach to the bone surface via αvβ3, αvβ5, α2β1, and αvβ1 integrins (Vaananen and Horton, 1995; Datta et al., 2008; Rauner et al., 2012; Plotkin and Bruzzaniti, 2019). At the bone/osteoclast surface, a ruffled border which is entirely surrounded by a sealing zone is formed, thereby creating an isolated resorption (Howship’s) lacuna (i.e., scalloped erosion) (Miyauchi et al., 1991; Mimura et al., 1994; Teitelbaum, 2000; Teitelbaum and Ross, 2003). Osteoclasts dissolve mineral (hydroxyapatite) and organic components (e.g., type I collagen) of the bone matrix in the resorption lacuna (Teitelbaum et al., 1995; Rauner et al., 2012). This resorption process is mediated by the secretion of hydrogen ions, to acidify the resorption compartment beneath osteoclasts and dissolve hydroxyapatite crystals (Blair et al., 1989; Teti et al., 1989). Hydrogen ions, supplied by the reaction of water and carbon dioxide and catalyzed by carbonic anhydrase II, are transported into the resorption lacuna by ATPases located in the ruffled border of osteoclasts (Baron, 1989; Mattsson et al., 1994; Li et al., 1999; Bruzzaniti and Baron, 2006; Hienz et al., 2015). Hydrochloric acid formed with chloride ions pumped into the resorption lacuna dissolves the mineralized bone matrix (Silver et al., 1988; Plotkin and Bruzzaniti, 2019). In addition, lysosomal enzymes (e.g., cathepsin K), bone-derived collagenases, and other proteinases (e.g., tartrate-resistant acid phosphatase) act in concert to mediate the resorption process (Bord et al., 1996; Gelb et al., 1996; Saftig et al., 1998; Boyle et al., 2003; Teitelbaum, 2007; Hienz et al., 2015). Osteoclast-mediated bone resorption, which takes a few (2-4) weeks during each remodeling cycle, results in Howship’s lacuna on the surface of trabecular bone and cylindrical Haversian canals in cortical bone (Bruzzaniti and Baron, 2006; Teitelbaum, 2007; Hienz et al., 2015). After one resorption lacuna is completed, the osteoclast cells die by apoptosis (Plotkin and Bruzzaniti, 2019) or move along the bone surface to resume resorption. This phase lasts approximately 8-10 days (Teitelbaum, 2007).
Systemic and local factors that stimulate bone resorption	Osteocytes as the major source of RANKL; thyroid hormones; PTH/PTHrP; calcitriol; glucocorticoids; growth factors (FGF, PDGF, EGF); TNF-α; colony-stimulating factors (M-CSF, GM-CSF); IL-1, -6, -7, -8, -11, -15, -17; PGE1, 2, 12; PGH2 (MacDonald, 1986; Dempster et al., 1993; Raisz, 1993; Kawaguchi et al., 1994, 1995; Nash et al., 1994; Holt et al., 1996; Lanske et al., 1999; Roodman, 1999; Lam et al., 2000; Compston, 2001; Ragab et al., 2002; Sher et al., 2004; Eijken et al., 2005; Dai et al., 2006; Zhang et al., 2008; Kini and Nandeesh, 2012; Rauner et al., 2012; Parra-Torres et al., 2013; Paiva and Granjeiro, 2017; Hachemi et al., 2018; Bellido and Gallant, 2019).

*RANK, receptor activator of nuclear factor kappa B; RANKL, receptor activator of nuclear factor kappa B ligand; GM-CSF, granulocyte-macrophage colony-stimulating factor; OPG, osteoprotegerin; TNF, tumor necrosis factor; IL, interleukin; DC-STAMP, dendritic-cell specific transmembrane protein; OC-STAMP, osteoclast stimulatory transmembrane protein; NFkB, nuclear-factor kappa B; TRAF6, TNF receptor-associated factor 6; TREM2, triggering receptor expressed on myeloid cells-2; OSCAR, osteoclast-associated receptor; DAP, DNAX-activating protein; FcRγ, Fc common receptor γ chain; FcRc, soluble Fc receptor from a group C streptococcus; Syk, spleen tyrosine kinase; PLC, phospholipase C; NFATc1, nuclear factor of activated T cell cytoplasmic 1; Tec, tyrosine protein kynase; AP, activator protein; Src, steroid receptor coactivator; PI3K, phosphatidylinositol 3-phosphate kinase; TRAP, tartrate-resistant acid phosphatase; LGR, leucine-rich repeat-containing G protein-coupled receptor; IFN, interferon; PTH, parathyroid hormone; PTHrP, PTH-related protein; FGF, fibroblast growth factor; PDGF, platelet-derived growth factor; EGF epidermal growth factor; M-CSF, macrophage colony-stimulating factor; PGE, prostaglandin E; PGH, prostaglandin H.*

The third ‘reversal’ phase connects osteoclastic bone tissue resorption and osteoblastic bone tissue formation (Delaisse, 2014) and lasts 7–14 days (Pettit et al., 2008; Hienz et al., 2015). After departure of the osteoclast from a cavity in bones undergoing resorption, which is a resorptive lacuna known as the Howship’s lacuna, bone lining cells occupy the Howship’s lacuna and clean it (Everts et al., 2002). The cleaning process occurs by enwrapping and digesting non-mineralized collagenous proteins protruding from the bone surface left by osteoclasts. This cleaning process is a requirement for the subsequent deposition of a first layer of collagen along the Howship’s lacuna (Everts et al., 2002). Four types of osteoclast-derived coupling factors stimulate bone formation during the reversal phase: (i) Matrix-derived factors including transforming growth factor-β, bone morphogenetic protein-2, platelet-derived growth factor, and insulin-like growth factors, which are released during bone tissue resorption, (ii) Osteoclast-secreted factors, including cardiotrophin-1, sphingosine-1-phosphate, collagen triple helix repeat containing 1, and complement factor 3a, (iii) Osteoclast membrane-bound factors such as EphrinB2 and Semaphorin D, and (iv) Structural changes brought about by the osteoclast on the bone tissue surface (Sims and Martin, 2014). Reversal cells originating from pre-osteoblast cells (Andersen et al., 2013) colonize the osteoclast-eroded surface and respond to osteoclast-derived messages and coupling factors along with fibroblast-like cells covering the surface of bone (known as bone lining cells), osteoblast precursors, and canopy cells (Delaisse, 2014; Sims and Martin, 2014; Lassen et al., 2017; Pirapaharan et al., 2019).

The fourth phase of the bone remodeling cycle is ‘formation,’ when mononucleate osteoblast cells synthesize new bone organic matrix formed by collagen fibers and non-collagenous proteins (e.g., bone sialoprotein, osteopontin, osteocalcin, proteoglycans) that later becomes surrounded and impregnated with mineral deposit mainly in the form of calcium hydroxyapatite. A summary of osteoblastogenesis, the roles played by osteoblasts during this last phase, and the fate of osteoblasts is described in Table 3.

**TABLE 3:** Osteoblasts and the bone formation phase.

Origin of osteoblasts	Differentiation stages: (i) From stem cell to mesenchymal (adult) stem cell; (ii) from mesenchymal stem cell to preosteoblast (immature); (iii) from preosteoblast to mature osteoblast (Datta et al., 2008).
Key factors involved in osteoblastogenesis	Hormones (such as PTH, glucocorticoids, estrogen, leptin, 1,25-dihy-droxyvitamin D3) (Datta et al., 2008; Mohanakrishnan et al., 2018; Arumugam et al., 2019; Plotkin and Bruzzaniti, 2019), growth factors (such as EGF, TGF-β, IGF) (Datta et al., 2008; Canalis, 2009; Plotkin and Bruzzaniti, 2019), local factors (such as the family of intracellular glycoproteins known as BMPs -2, -4, -6, -7) (Shore et al., 2006; Wutzl et al., 2010), members of the Wnt family in a paracrine/autocrine fashion (Bodine and Komm, 2006), Sonic and Indian hedgehogs (Maeda et al., 2007; Guan et al., 2009), cell-to-cell communication through receptors (such as Notch, Ephrin-Ephrin) and connexins (e.g., Cx43) (Plotkin and Bruzzaniti, 2019).
Key signaling events involved in the canonical Wnt/β-catenin pathway	Wnt proteins bind to FZD receptor and its co-receptor (e.g., LRP4, LRP5, LRP6). CK1α then phosphorylates Dvl and in turn the complex Dvl-Frat1-axin-LRP5/6-FZD is formed. These events result in GSK3β inhibition, thereby avoiding modification (degradation, phosphorylation) of β-catenin. The stable β-catenin is then translocated to the nucleus to activate transcription factors (e.g., TCF, LEF), thus inducing the transcription of Wnt target genes (e.g., osteoprotegerin) (Datta et al., 2008; Plotkin and Bruzzaniti, 2019). Wnt signaling is regulated by a variety of molecules at the levels of extracellular inhibition of Wnt ligands or LRP4/5/6, co-receptors, intracellular signaling, and transcription (Gong et al., 2001; Boyden et al., 2002; Tian et al., 2003; Logan and Nusse, 2004; Semenov et al., 2005; Datta et al., 2008; Chen et al., 2019). Besides the canonical Wnt/β-catenin pathway, Wnt ligands can also activate other different signaling cascades (such as the Wnt-Ca(II), planar cell polarity, and protein kinase A pathways).
Key signaling events involved in the BMPs pathway	BMPs (e.g., BMP-2, BMP-7) as well as other signaling pathways (e.g., members of the Wnt pathway, TGFβ1, Indian hedgehog, notch, ephrin [Huang et al., 2007; Datta et al., 2008; Rauner et al., 2012; Plotkin and Bruzzaniti, 2019]) converge to regulate the expression of runx2 and others (such as β-catenin [Krishnan et al., 2006], osterix [Kim et al., 2006], msx2 [Liu et al., 1999; Satokata et al., 2000], NFATc1 [Koga et al., 2005], ATF4 [Tozum et al., 2004], Dlx3/5/6 [Harris et al., 2003], FGFR3, FGFs [e.g., -2, -9, -18], Phex, NFAT2, ALP) (Paiva and Granjeiro, 2017). Specifically, the expression of runx2, which is the master transcription factor, is controlled by Twist and menin-1, TAZ, and post-translational modifications. Also, Runx2 phosphorylation under TGF-β1 stimulation occurs at three serine amino acids (Arumugam et al., 2018). Osterix may interact with NFAT2, which participates in regulating gene transcription (e.g., for osteopontin, osteoclacin, osteonectin) (Paiva and Granjeiro, 2017).
Roles played by osteoblasts	Once osteoclasts have created a resorption cavity and detached from the bone surface, osteoblasts move into the cavity to initiate bone formation (Datta et al., 2008). Osteoblasts synthesize and lay down new unmineralized bone matrix (osteoid), which is subsequently mineralized (e.g., forming hydroxyapatite) over a period of about 20 days. Osteoblasts also synthesize and secrete the bone matrix proteins osteopontin, osteocalcin, bone sialoprotein, proteoglycans, and alkaline phosphatase (Baron, 1989; Roach, 1994; Ducy et al., 2000; Datta et al., 2008; Hienz et al., 2015). Why is the synthesis of non-collagen proteins necessary? These non-collagenous bone matrix proteins help to coordinate matrix mineralization and are essential for cellular adhesion (such as chemoattractant activity by osteocalcin), and regulation of cell activity (such as the osteopontin- and osteonectin-displayed cell activities) during coupling of bone resorption and formation (Robey, 1989; Raynal et al., 1996; Hienz et al., 2015). There is another function of osteoblasts that is worth highlighting. Osteoblasts also inhibit the ability of osteoclasts to degrade osseous tissue (Datta et al., 2008).
Stimulators of osteoblast functions	The increased formation of osteoid to build bone is stimulated by hormones (such as the pituitary-secreted growth hormone, sex hormones [estrogens and androgens], and thyroid hormone) (Kini and Nandeesh, 2012). Other factors that have stimulating effect on bone formation are insulin, vitamin D metabolites, IGF-I, IGF-II, TGF-β, BMP-2, BMP-4, BMP-6, BMP-7, IL-13, IFN, and OPG (Baylink et al., 1993; Cohick and Clemmons, 1993; Fraher, 1993; Rosen and Donahue, 1998; Yamaguchi et al., 2000; Canalis et al., 2003; Lovibond et al., 2003; Datta et al., 2008; Tang et al., 2009; Ruan et al., 2010; Kini and Nandeesh, 2012; Xian et al., 2012; Hienz et al., 2015).
Osteoblast fate	Bone-forming osteoblasts become encased in the mineralized matrix surrounding them, turning into osteocytes that gradually stop synthesizing osteoid (i.e., the newly formed unmineralized organic bone matrix) (Datta et al., 2008; Rauner et al., 2012). Osteocytes are evenly distributed throughout the bone matrix which enables contact with osteoblasts and vasculature (Kamioka et al., 2001; Plotkin et al., 2002; Zhao et al., 2002; Plotkin et al., 2008). Osteocytes not only facilitate mechanosensation as described in Table 1, but also control bone structure (amount and quality) through mineralization inhibitors such as dentin matrix protein-1, fetuin-A, and Wnt inhibitor (Poole et al., 2005; Feng et al., 2006; Coen et al., 2009; Liu et al., 2009; Rauner et al., 2012). Although it was thought that osteocytes remain inactive until the next bone remodeling cycle (Mikuni-Takagaki, 1999; Kamioka et al., 2001; Zhao et al., 2002; Knothe-Tate et al., 2004; Datta et al., 2008), it is now accepted that osteocytes constantly remodel the surrounding extracellular matrix (Yee et al., 2019). Another fate of osteoblasts is to become bone lining cells, which cover the freshly formed endosteal bone surface thus forming a physical barrier to avoid the process of osteoclast adhesion and bone resorption.

*PTH, parathyroid hormone; EGF, epidermal growth factor; TGF transforming growth factor, IGF, insulin-like growth factor; BMP, bone morphogenetic protein; LRP, low-density lipoprotein receptor-related protein; FZD, seven-span transmembrane receptor protein Frizzled; CK, casein kinase; Dvl, disheveled; GSK, glycogen synthase kinase; TCF, T cell factor; LEF, lymphoid enhancer binding factor; runx2, runt-related transcription factor 2; osterix, Sp7 transcription factor; msx2, homeobox factor; NFAT, nuclear factor of activated T cells; ATF, activating transcription factor; Dlx, distal-less homeobox; FGF, fibroblast growth factor; FGFR, fibroblast growth factor receptor; Phex, phosphate-regulating neutral endopeptidase; ALP, alkaline phosphatase; TAZ, tafazzin; IL, interleukin; IFN, interferon; OPG; osteoprotegerin; Wnt, Wingless-type MMTV integration site family.*

While bone formation surpasses resorption during childhood, bone formation and resorption are in balance during young adulthood. However, an unbalanced bone loss occurs with aging (Datta et al., 2008; Rauner et al., 2012; Brandi and Piscitelli, 2013) and could predispose an individual to skeletal disorders including: (i) inflammatory bone loss in periodontal disease, (ii) arthritis (stimulation of bone resorption and inhibition of bone formation by prostaglandins and cytokines), (iii) osteoporosis (bone resorption outpaces bone formation), (iv) hyperparathyroidism and hyperthyroidism (greatly increased rate of bone resorption and formation), (v) Paget’s disease (increased and abnormal [shape, weakness, and brittleness] bone formation), (vi) osteomalacia (delayed/defficient bone mineralization), and (vi) osteopetrosis (failure of osteoclasts to resorb bone) (Roodman et al., 1992; Delmas, 1995; Gallagher, 1997; Mills and Frausto, 1997; Raisz, 1997; Charles and Key, 1998; Schneider et al., 1998; Siris, 1998; Kini and Nandeesh, 2012).

## Matrix Metalloproteinases: Modulators of Bone Remodeling

**FIGURE 1:**
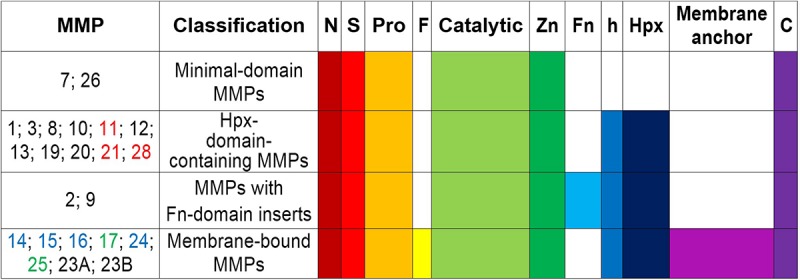
Schematic structure and classification of matrix metalloproteinases. S, amino-terminal signal sequence; Pro, pro-peptide; Zn(II)-binding site; h, hinge region; Hpx, hemopexin; FN, collagen-binding type II repeats of fibronectin; F, furin; MT-MMPs, membrane-type MMPs; N, N-terminus; C, C- terminus. MMPs -11, -21, and -28 (all in red) contain a Furin-like cleavage domain. MMPs -17 and -25 (both in green) contain a glycophosphatidyl inositol-anchoring sequence. MMPs -14, -15, -16, and -24 (all in blue) comprise a transmembrane domain with a cytosolic tail. MMP23A and MMP23B lack the signal peptide, the cysteine-switch motif and the hemopexin-like domain, but they contain a unique cysteine-rich domain, an immunoglobulin-like domain and an N-terminal type II transmembrane domain (Velasco et al., 1999).

Matrix metalloproteinases are a family of at least 24 highly homologous, multi-domain enzymes (Figure 1) with the capacity to degrade virtually all extracellular matrix components including collagen, aggrecan, elastin, and fibronectin (Lu et al., 2011; Fernandez-Patron et al., 2016).

All MMP family members are synthesized as catalytically inactive (latent) pro-enzymes (pro-MMPs) that contain a: signal N-terminal peptide sequence (∼20 amino acids), pro-peptide domain (∼80 amino acids), catalytic domain (approximately 160 amino acids), hinge (linker peptide) region of variable length (10–30 amino acids), and a hemopexin-like C-terminal domain (Hpx) (∼210 amino acids). The smallest MMPs (MMP-7 and MMP-26) lack the hinge and hemopexin domains, and therefore exhibit a reduced affinity for gelatin. MMP-23 has unique domains (such as the cysteine array, IgG-like domain, interleukin-1 type II receptor-like domains) instead of the hemopexin domain (Massova et al., 1998; Pei et al., 2000; Bode and Maskos, 2003; Visse and Nagase, 2003; Nagase et al., 2006; Piccard et al., 2007; Lopez-Otin et al., 2009; Bonnans et al., 2014; Vandooren et al., 2014; Vandenbroucke and Libert, 2014; Cui et al., 2017). The amino-terminal signal peptide targets the pro-MMPs to the rough endoplasmic reticulum, whereas the C-terminus harbors a cysteine residue and a furin cleavage site (PRCGXPD), both of which are important for conversion into the mature, active enzyme (Bonnans et al., 2014). Presence of an intact pro-peptide accounts for the latency of pro-MMPs, which can be overriden through the activation of a “cysteine-switch” mechanism (Van Wart and Birkedal-Hansen, 1990). The pro-peptide contains a cysteine residue that prevents catalytic activity when it is coordinated with a Zn(II)-ion in the catalytic domain (Springman et al., 1990; Van Wart and Birkedal-Hansen, 1990). The cysteine-Zn(II) interaction can be disrupted by alkylating compounds such as the organomercurial 4-aminophenylmercuric acetate as well as by serine proteases and other MMPs such as membrane-type MMPs, which act at the cell surface to which they anchor through their transmembrane domain/short cytoplasmic tail or by glycosylphosphatidylinositol linkage (Bonnans et al., 2014). MMP autolysis is another mechanism of activation mediated by allosteric perturbation of the inactive proenzyme (Springman et al., 1990; Van Wart and Birkedal-Hansen, 1990; Pei and Weiss, 1995; Pei et al., 2000; Meng et al., 2016). The catalytic domain harbors the Zn(II)-binding motif HEXXHXXGXXH, a catalytic Zn(II), a structural Zn(II), specific pockets related to specificity (S1, S2,…Sn and S1′, S2′,…Sn′) and coordinated Ca(II) ions which confer stabilization. The catalytic Zn(II) is coordinated by three histidine residues (Bode and Maskos, 2003; Bonnans et al., 2014; Vandenbroucke and Libert, 2014). The hinge domain is flexible and mediates interactions with substrates, cell-surface proteins, and tissue inhibitors (Cui et al., 2017; Liu and Khalil, 2017). The hemopexin domain modulates substrate recognition and specificity, binding to cell-surface receptors and inhibitors, activation of MMPs, and cellular MMP internalization for degradation (Visse and Nagase, 2003; Nagase et al., 2006; Piccard et al., 2007).

Matrix metalloproteinases expression and activity are tightly regulated at various levels: gene transcription, translation and secretion of the inactive enzyme precursor, proteolytic activation of the zymogen, spatial localization, interaction with specific extracellular matrix proteins, and inhibition by endogenous inhibitors (such as tissue inhibitors of MMPs [TIMPs 1-4], α2-macroglobulin, and human fibrinogen) (Sottrup-Jensen, 1989; Overall et al., 1991; Kusano et al., 1998; Zeng et al., 1998; Sternlicht and Werb, 2001; Han et al., 2003; Greenlee et al., 2007; Clark et al., 2008; Fanjul-Fernandez et al., 2010; Hadler-Olsen et al., 2011; Arpino et al., 2015; Sarker et al., 2019). Despite their similar names, TIMPs 1-4 exhibit large differences in their primary sequence, tissue expression, transcriptional regulation and in their inhibitory spectrum (Brew et al., 2000). In bone, TIMP-2 and TIMP-3, unlike TIMP-1, are effective inhibitors of the membrane-type MMPs (e.g., MMP-14), while TIMP-3 displays the broadest inhibitory actions of all TIMPs against metalloproteinases. Unlike TIMP-1, -2, and -4, which are soluble, TIMP-3 has basic amino acid residues in its C- and N-termini through which TIMP-3 attaches to heparan and chondroitin sulfate in the extracellular matrix and inhibits both MMPs and members of ‘a disintegrin and metalloproteinase’ (ADAM) and ‘a disintegrin and metalloproteinase with thrombospondin domains’ (ADAMTS) family including ADAM-17 and ADAMTS-4/-5 (Porter et al., 2005; Javaheri et al., 2016). Deficiency of tissue inhibitors (TIMP-1, -2, or -4) has minor impact on bone phenotype. However, both *Timp3* deficiency and transgenic overexpression alters craniofacial bones of endochondral and intramembranous origins in mice, while the growth plates appear normal in these mice (Javaheri et al., 2016). Paradoxically, mice deficient in RECK (an MMP inhibitor anchored on the cell membrane with inhibitory actions against MMP-2, -9, and -14 and ADAM-10) die *in utero* displaying a perturbed extracellular matrix organization (Javaheri et al., 2016).

These observations suggest that bone remodeling may not be solely defined by the balance/imbalance between MMPs and TIMPs. Rather, other molecules expressed and released in the settings of bone physiology and pathology such as RECK (Paiva and Granjeiro, 2014) and some acute phase reactants (alpha 2-macroglobulin, fibrinogen) may regulate/dysregulate MMP activity in inflammatory conditions thus perturbing the normal bone remodeling process (Cook et al., 2018; Sarker et al., 2019). A consequence implied by the latter notion is that MMPs, ADAMs and ADAMTS molecules may be released from bone or non-bone tissues to influence bone remodeling through autocrine and paracrine actions. In other words, MMPs likely circulate bound to non-classical inhibitors (such as acute phase reactants) being recruited to sites of active bone remodeling, where local substrates act as chemoattractants and local activators (other proteases, reactive oxygen species) activate them.

The aforementioned levels of regulation effectively dissociate MMP expression from MMP activity (e.g., since overexpression of endogenous MMP inhibitors would effectively reduce MMP activity). Current biochemical techniques for assessing MMP activity are non-reliable. However, as research requires a proxy, MMP expression is often used as a surrogate (albeit incorrectly) for MMP activity. There remains an urgent need for highly sensitive, specific, and robust methods for assessing the activity potential of individual MMPs such that therapeutic strategies can be designed to specifically reduce the activity of overactive MMPs (i.e., those whose activity levels are above baseline) or to increase the activity of underactive MMPs (i.e., those whose activity levels are below baseline).

### Roles of MMPs Associated to Bone Development and Remodeling

The biochemical actions of MMPs are intimately linked to their cells of origin. Table 4 describes cell-specific roles of MMPs in physiological bone remodeling. Osteoclast-mediated bone resorption in calvaria and long bones requires normal enzymatic activity of MMPs and cysteine proteinases such as cathepsin K whose deficiency impairs bone remodeling (Everts et al., 1999; Delaisse et al., 2003). This is evidenced in osteoclasts from patients with pycnodysostosis (an osteopetrosis-like bone disease related to loss-of-function mutations in the cathepsin K gene) and osteoclasts from cathepsin K-deficient mice which are unable to efficiently digest organic bone matrix, resulting in large, mineral-free areas of bone matrix (Everts et al., 1998, 2009). Cysteine proteinases synthesized and used by the different osteoclasts for bone matrix digestion (Everts et al., 2006) can degrade intramembranous bones as well as osteoclast-derived MMPs (Everts et al., 2009). Cysteine proteinases are secreted to act in the low pH environments formed by osteoclasts in the resorption sites, with MMPs degrading the rest of the bone matrix when the pH increases (Everts et al., 1998) as well as contributing to the digestion of fibrillar, non-mineralized collagen in Howship’s lacunae abandoned by osteoclast cells (Everts et al., 2002). These complementary and overlapping contributions of the MMP and cysteine proteinase families make the process of bone tissue remodeling both complex and robust.

**TABLE 4:** Specific roles of MMPs under physiological conditions in bone remodeling.

**Entity**	**MMP**	**Role**	**References**
Cartilage and bone cells	Network of multiple MMPs (mainly widely expressed MMP-2, -7, -9, -12, -13, -14, -16)	Maintain bone and cartilage health by their normal proteolytic activity.	Everts et al., 1992; Meikle et al., 1992; Mattot et al., 1995; Apte et al., 1997; Johansson et al., 1997; Bord et al., 1998; Jimenez et al., 1999; Filanti et al., 2000
		Control bone tissue remodeling at the levels of osteocyte viability and activities, osteoclast recruitment and function, bone matrix solubilization, coupling of bone resorption and formation, osteoblast recruitment and survival, cell-extracellular matrix interaction, and cell–cell interaction.	Blavier and Delaisse, 1995; Bord et al., 1998; Engsig et al., 2000; Hou et al., 2004; Inada et al., 2004; Karsdal et al., 2004; Holmbeck et al., 2005; Kasper et al., 2007; Manduca et al., 2009; Lu et al., 2010; Ortega et al., 2010; Tang et al., 2012; Madsen et al., 2013; Lozito et al., 2014; Almalki and Agrawal, 2016
		Regulate the bioavailability of soluble RANKL, thereby promoting the formation of multinucleated osteoclast cells, acquisition of osteoclast-specific differentiation markers, binding of osteoclasts to bone surfaces, promotion of osteoclast survival, and stimulation of bone resorption.	Bellido et al., 2019
Mesenchymal stem cells	Network of multiple MMPs, tissue inhibitors of MMPs and RECK	(i) Modulates the commitment and differentiation of mesenchymal stem cells. (ii) Impacts osteoblastic migration, spreading, and differentiation.	Kasper et al., 2007; Lu et al., 2010; Lozito and Tuan, 2011; Egea et al., 2012; Almalki and Agrawal, 2016; Mahl et al., 2016
	MMP-16	Controls mesenchymal stem cells viability.	Paiva and Granjeiro, 2017
	MMP-2 and MMP-9	Promote the directional migration of bone marrow mesenchymal stem cells.	Lv et al., 2017
Osteocytes	MMP-2, MMP-13 and MMP-14	Modulate the formation of the osteocyte canalicular network.	Barthelemi et al., 2012
	MMP-13	Regulates the remodeling of the osteocyte lacunar-canalicular network in mid-cortical bone matrix, which is critical for the active maintenance of bone quality (matrix composition, organization, fracture resistance).	Tang et al., 2012; Alliston, 2014
	MMP-14	Essential for cell adhesion, invasion, and cell-cell communication events.	Hughes et al., 1994; Paiva and Granjeiro, 2017
Osteoclasts	MMP-9	Participates in cell recruitment (by generating collagen-derived endostatin which prevents osteoclast chemotaxis), survival (e.g., by activating pro-TNF-α), adhesion (e.g., by cleaving intercellular adhesion molecule-1), as well as in degradation of cytokines important to osteoclastogenesis such as IL-1β.	Gearing et al., 1995; Ito et al., 1996; Ferreras et al., 2000; Fiore et al., 2002
	MMP-12	Modulates the interaction between osteoclasts and bone matrix through multiple mechanisms including: (i) cleavage of osteopontin, vitronectin, bone sialoprotein and osteonectin, (ii) activation of TNF-α, (iii) generation of endostatin from collagen, and (iv) digestion of urokinase-type plasminogen activator receptor/uPAR.	Koolwijk et al., 2001; Hou et al., 2004; Paiva and Granjeiro, 2017
	MMP-14	Sheds CD14 receptor to impinge on osteoclast adhesion and migration as well as being involved in monocyte/macrophage fusion (e.g., by modulating the Rac1 pathway).	Kajita et al., 2001; Vivinus-Nebot et al., 2004; Gonzalo et al., 2010
	The CD44/MMP-9/MMP-14 axis	Mediates pro-MMP-9 activation on the osteoclast membrane thereby modulating osteoclast migration in bone tissue resorption.	Chellaiah and Ma, 2013
	MMP-14 and MMP-7	Promote RANKL availability, which implicates the RANK/RANKL/osteoprotegerin axis in osteoclast maturation and activation.	Lynch et al., 2005; Hikita et al., 2006; Aiken and Khokha, 2010
Bone matrix	MMP-1, -2, -8, -9, -13, -14, and -15	Necessary for extracellular matrix turnover.	Paiva and Granjeiro, 2017
	MMPs -2, -3, -7, -9, -12, -14	Cleave and regulate bone matrix-associated non-collagenous proteins (such as osteonectin, vitronectin, osteopontin, bone sialoprotein) as well as cell membrane- and matrix-anchored latent growth factors.	Sasaki et al., 1997; Agnihotri et al., 2001; Sage et al., 2003; Lindsey et al., 2015
	MMP-14	The collagen fragments produced by MMP-14 are endocytosed via uPARAP/Endo180 for total lysosomal degradation.	Lafleur et al., 2006; Lee et al., 2006; Messaritou et al., 2009
Osteoblasts	MMP-2	Critical for osteoblast differentiation and survival.	Paiva and Granjeiro, 2017
	MMP-14	Serves to preserve osteoblast survival once osteoblasts have stopped the synthesis of new bone matrix, thus aiding in the transition from osteoblasts to osteocytes.	Karsdal et al., 2004
Bone remodeling	MMPs from osteoblasts and bone lining cells	Preceding osteoclast adhesion and resorption, MMPs participate in the cleavage of organic matrix (such as cathepsin-cleaved collagen and non-collagenous proteins).	Holliday et al., 1997; Stahle-Backdahl et al., 1997; Yamagiwa et al., 1999; Paiva and Granjeiro, 2017
	MMP-13	Active in regulating bone mass through osteoblasts, and forming osteocyte canalicular network.	Page-McCaw et al., 2007; Barthelemi et al., 2012
	MMP-14/CD44	Activates Pro-MMP-9 on osteoclast membrane surface during osteoclast recruitment, adhesion, resorption and migration.	Paiva and Granjeiro, 2017

*MMPs, matrix metalloproteinases; RECK, reversion-inducing cysteine-rich protein with Kazal motifs; TNF, tumor necrosis factor; IL, interleukin; Rac1, Ras-related C3 botulinum toxin substrate 1 pathway; RANK, receptor activator of nuclear factor kappa B; RANKL, RANK ligand; uPARAP/Endo180, endocytic collagen receptor of collagen and collagen fragments for degradation in the lysosomes.*

The involvement of MMPs in bone remodeling has become clear with the aid of animal models such as MMP-deficient mice, which show a variety of bone abnormalities (Table 5). Impaired bone tissue remodeling in *Mmp2^–/–^* mice (Table 5, row 2) is characterized by a reduced number of osteoblasts and osteoclasts, disruption of the canicular network exacerbating osteocyte death, disruption of the MMP-2-osteopontin-bone sialoprotein axis, and promotion of osteolysis (Martignetti et al., 2001; Inoue et al., 2006; Mosig et al., 2007; Malaponte et al., 2016). MMP-9-deficient mice show alterations in cartilage-bone replacement during endochondral ossification (Vu et al., 1998) (Table 5, row 3). This phenotype may be explained by an inefficient degradation of the cartilage matrix, which leads to a diminished bioavailability of extracellular matrix-derived vascular endothelial growth factor and consequently effects osteoclasts and endothelial cells movement into the cartilage (Ortega et al., 2010). Bone tissue modeling and remodeling processes are altered in MMP-13 deficient mice (Table 5, row 4) (Inada et al., 2004; Stickens et al., 2004; Ortega et al., 2005). MMP-14 deficiency (Table 5, row 5), which is associated with high lethality, results in the most drastic skeletal phenotype among MMP-deficient mice (Holmbeck et al., 1999; Zhou et al., 2000). Double gene-deficient mice lacking at least one MMP gene have been engineered and their bone phenotype have been studied. For instance, double-knockout mice lacking MMP-2 and uPARAP/Endo180 (endocytic receptor of collagen and collagen fragments for degradation in the lysosomes) show reduced bone mineral density, short long bones, and poor trabecular bone quality (Madsen et al., 2013). MMP-8 and MMP-13 double-deficient mice have abnormal growth plate as well as augmented metaphyseal trabecular bone mineral density (Inada et al., 2001, 2002; Stickens et al., 2004). Double knockout mice lacking MMP-9 and MMP-13 exhibit expanded growth plates, disorganized hypertrophic chondrocyte zone, increased number of end-differentiated hypertrophic cells, and delayed formation of the bone marrow cavity (Kennedy et al., 2005; Paiva and Granjeiro, 2014). The bone phenotype of mice with a double knockout for MMP-14 and MMP-2 reassembles that of MMP-14-deficient mice (Oh et al., 2004). MMP-14 and MMP-16 double-knockout mice develop a bone phenotype that affects ossification (intramembranous and endochondral) and is characterized by severe irregularities, including (i) high mortality associated to developmental defects, (ii) noticeable craniofacial malformations such as cleft palate, thinner cranial vault bones, deficiently developed parietal, as well as frontal and nasal bones, (iii) altered growth plate, and (iv) cortical bone shortening (Paiva and Granjeiro, 2014). MMP-14 and uPARAP/Endo180 double-knockout mice die soon after birth (Wagenaar-Miller et al., 2007). As listed in Table 6, MMP activity contributes to numerous bone pathologies including arthritis, osteoporosis, osteonecrosis, periodontitis, sinonasal osteitis, degenerated lumbar disk tissues, and bone cancer metastasis (Aiken and Khokha, 2010; Koskinen et al., 2011; Mittal et al., 2016; Rose and Kooyman, 2016; Lazarus et al., 2017; Paiva and Granjeiro, 2017; Tauro and Lynch, 2018; Zhang et al., 2018). The roles played by MMPs in these pathologies are influenced by non-matrix proteins such as TIMPs, transforming growth factor, vascular endothelial growth factor, bone morphogenic proteins, activated protein C, and the Wnt [Wingless-type MMTV integration site family]/β-catenin (Table 7).

**TABLE 5:** Selected skeletal phenotypes associated to MMP deficiency in mice.

**Genotype**	**Phenotype**	**References**
*Mmp2*^–/–^	MMP-2 knockout (vs. wild-type) mice show: (i) craniofacial defects (such as shorter and broader snouts, hypertelorism, smaller jaws, dome-shaped and taller skulls), (ii) severe arthritis and joint contractures (even in young mice) with articular cartilage destruction and erosion of the underlying bone surface, (iii) joint pathology with increased cellular infiltration and proteoglycan depletion in antigen-induced arthritis, (iv) diminished bone integrity (such as long bones with osteopenia, fractured tibiae), (v) anomalous bone development (e.g., reduced number of long bones, decreased femur and tibia length in adult mice, calvarial bones with a greater [48%] thickness by 55 weeks of age, trabecular bone with fewer osteocytes), (vi) progressive decrease in bone mineral density and increase in bone porosity (characterized by e.g., low trabecular connectivity density, reduced mineral-collagen relation, thinner diaphyseal cortex, less nanoindentation modulus), (vii) increased number of empty lacunae as the mice aged (e.g., about 3-fold by 55 weeks of age), (viii) loss of the canalicular network architecture in calvariae and slighter in long bones, and (ix) presumably expression of bone sialoprotein (which increases osteoblast differentiation and activity) and osteopontin (which increases osteoclast activity).	Inoue et al., 2006; Mosig et al., 2007; Lieu et al., 2011; Nyman et al., 2011; Madsen et al., 2013
*Mmp9*^–/–^	MMP-9 knockout (vs. wild-type) mice show: (i) long bones (e.g., metatarsals) with increased (e.g., 4-8-fold for 3 weeks old mice) hypertrophic (cartilage) zones, (ii) 10% shorter long bones, which is the only remaining phenotype in older MMP-9 deficient mice, (iii) irregularly shaped bone spicules, (iv) delayed endochondral ossification, (v) expanded zone of hypertrophic chondrocytes in the growth plate, (vi) reduced vascular invasion into the hypertrophic cartilage, (vii) slowed apoptosis of hypertrophic chondrocytes, (viii) impaired osteoclast/condroclast recruitment, (ix) anomalous growth in trabecular bone mass, and (x) improved connectivity density of the tibia trabeculae. This phenotype eventually resolve, resulting in correction of bone growth defects after approximately 4 weeks of age.	Vu et al., 1998; Ortega et al., 2003; Nyman et al., 2011; Kojima et al., 2013
*Mmp13*^–/–^	*Mmp13^–/–^* (vs. *Mmp13*^±^) mice embryos show: (i) progressive changes in the embryonic growth plates (e.g., increased length which persisted in adults), (ii) delayed endochondral ossification, (iii) augmented metaphyseal trabecular bone mass as the mice aged (e.g., 3 months old), (iv) diminished resistance to fracture in long bones, (v) delay in fracture repair, (vi) defective vascular penetration and chondroclast attraction to the fracture callus, (vii) noticeable expression of collagen type X, osteopontin, and VEGF by hypertrophic chondrocytes.	Inada et al., 2001; Inada et al., 2002; Inada et al., 2004; Stickens et al., 2004; Kosaki et al., 2007; Tang et al., 2012; Singh et al., 2013
*Mmp14*^–/–^	MMP-14 knockout (vs. wild-type) mice show: (i) progressive disturbances (e.g., smaller body size and weight, very high postnatal mortality), possibly caused by deprived feeding and therefore malnutrition, (ii) craniofacial dysmorphism in surviving mice (e.g., short snout, hypertelorism, dome-shaped skull, orbital protrusions, unclosed cranial sutures), (iii) incomplete cartilage remodeling, (iv) impaired formation of secondary ossification centers in the epiphyses, (v) ankylosis resulting from joints with arthritis and other factors (e.g., greater vascularity of the ligaments and tendons, overgrowth of hypercellular and wrongly vascularized synovial tissue), (vi) augmented bone resorption, (vii) osteopenia, (viii) osteoporosis, (ix) dwarfism, (x) mesenchymal stem cells commitment to chondrogenesis and adipogenesis instead of osteogenesis.	Holmbeck et al., 1999; Zhou et al., 2000; Holmbeck et al., 2003
*Mmp16*^–/–^	MMP-16 knockout (vs. wild-type) mice show shorter size associated with reduced viability of mesenchymal cells in bone tissues.	Shi et al., 2008; Loffek et al., 2011

*MMP, matrix metalloproteinase; VEGF, vascular endothelial growth factor.*

**TABLE 6:** Involvement of MMPs in bone pathologies.

**MMP**	**Reported involvement**	**References**
MMP-1	Abundant in the diaphysis and metaphyses of long bones being upregulated in arthritis.	Gack et al., 1995; Wu et al., 2008; Rose and Kooyman, 2016
MMP-2	Required for maintenance of bone mineral density and strength and in bone development (e.g., by affecting intramembranous and endochondral ossification); however, deregulated MMP-2 expression is observed in the settings of metabolic syndrome, osteoporosis, osteonecrosis of the jaws, ligamentum flavum degeneration in lumbar spinal canal stenosis, as well as in bone pre-metastatic niche formation.	Duerr et al., 2004; Suh et al., 2004; Tester et al., 2004; Durie et al., 2005; Lynch, 2011; Fernandez-Patron et al., 2016; Rose and Kooyman, 2016; Sugimoto et al., 2018
MMP-3	Overexpressed in osteoarthritis (in cartilage and the synovium) and also acts on primary tumor growth.	Okada et al., 1992; Tester et al., 2004; Lynch, 2011; Paiva and Granjeiro, 2017
MMP-8	Modulates human dentin and remodeling, but its deregulation may exacerbate periodontitis although it may be protective against inflammatory arthritis.	Sulkala et al., 2007; Cox et al., 2010; Mauramo et al., 2018
MMP-9	Participates in chondrocyte biology; specific processes in which the enzyme is involved are apoptosis of hypertrophic chondrocytes present *in utero*, bone development (e.g., by being highly active to angiogenesis in the growth plate), strength and toughness of bone, as well as the regulation of gene pathways responsible for osteoclastogenesis). In turn, MMP-9 overexpression contributes to sinonasal osteitis, rheumatoid arthritis, and degenerated lumbar disk tissues.	Vu et al., 1998; Liang et al., 2016; Mittal et al., 2016; Ahrens et al., 1996; Li et al., 2017
	Osteoporotic bone (vs. normal bone) tissues express higher MMP-9 levels.	Zhao et al., 1997
	Involved in secondary (metastatic) breast cancer in the bone (e.g., by promoting angiogenesis, regulating VEGF bioavailability, contributing to bone remodeling) or prostate cancer (e.g., by influencing bone osteoblastic and osteoclastic activity).	Bergers et al., 2000; Mannello et al., 2005; Pego et al., 2018
MMP-13	Required for bone development; it participates in the transition from cartilage to bone at the growth plates of long bones and in the remodeling of bone spicules. In turn, MMP-13-mediated degradation of articular cartilage exacerbates osteoarthritis.	Inada et al., 2004; Stickens et al., 2004; Page-McCaw et al., 2007; Holmbeck et al., 1999; Mittal et al., 2016; Rose and Kooyman, 2016
	In linking osteoarthritis to metabolic syndrome, the presence of adiponectin positively correlates with the presence of membrane-expressed PGE synthase and MMP-13.	Francin et al., 2014
	Overexpressed in congenital spondyloepiphyseal dysplasia which results in early development of osteoarthritis.	Rose and Kooyman, 2016
	In addition to typical bone collagen matrix degradation, MMP-13 regulates bone resorption in periodontal disease through osteoclast differentiation (by inactivating galectin-3, an inhibitor of osteoclastogenesis) and osteoclast activation (by activating osteoclast-secreted pro-MMP-9 and favoring RANKL and TGF-β1 signaling).	Nannuru et al., 2010; Pivetta et al., 2011; Cavalla et al., 2017
	In breast cancer resulting from bone metastasis, MMP-13 deregulation may alter osteoblast morphology and bone resorption through differentiation of pre-osteoclasts, osteoclast activation, and osteolysis.	Stickens et al., 2004; Page-McCaw et al., 2007; Shah et al., 2012
MMP-14	Contributes to bone development (endochondral and intramembranous ossification) and remodeling. Extracellular matrix remodeling by MMP-14 influences cell shape inducing the formation of a complex between MMP-14 and beta1-integrin, which activates the Rho/GTPase cascade leading to nuclear translocation of YAP and TAZ – this series of signaling events is necessary for mesenchymal stem cells commitment during development. Palmitoylation (i.e., addition of 16-carbon palmitate to proteins) enables MMP-14 to anchor to cell membrane. This post-translational modification of MMP-14 has a major impact on bone development and bone tissue metabolism likely through influencing MMP-14 correct membrane localization and also decreasing the expression of osteocalcin and vascular endothelial growth factor in osteoblasts and chondrocytes. In turn, MMP-14 is critical for osteoclast resorption thus contributing to the pathogenesis of osteoporosis.	Holmbeck et al., 1999, 2003; Zhou et al., 2000; Liao et al., 2004; Hienz et al., 2015; Tang et al., 2013; Paiva and Granjeiro, 2014; Song et al., 2014
	Involved in bone cancer metastasis acting alongside MMP-1 and MMP-11.	McGowan and Duffy, 2008; Rowe and Weiss, 2009; Paiva and Granjeiro, 2017
MMP-3 and MMP-9	Contribute to cartilage endplate degeneration.	Zhang et al., 2018
MMP-2, MMP-9, and MMP-13	In experimental glucocorticoid-induced osteoporosis and osteocytic osteolysis, these three enzymes are upregulated in the trabecular bone of the metaphysis whereas MMP-2 and MMP-13 are expressed in the cortical bone diaphysis.	Sun et al., 2016

*VEGF, vascular endothelial growth factor; PGE, prostaglandin E synthase; RANKL, receptor activator of NF-kappa B ligand; TGF transforming growth factor; MMP, matrix metalloproteinase; YAP, Yes-association protein; TAZ, transcriptional coactivator with PDZ-binding motif.*

**TABLE 7:** Interactions of MMPs with other proteins in bone development/remodeling.

**Protein**	**Effect on the partner**	**Effect on bone development/remodeling**
TIMPs	Inhibit all MMPs	Control bone resorption and formation (Bord et al., 1999; Sobue et al., 2001; Huang et al., 2002; Geoffroy et al., 2004; Haeusler et al., 2005; Sahebjam et al., 2007; Shen et al., 2010; Miller et al., 2017).
MMP-2/MMP-9	Control TGF-β (bioavailability and bioactivity)	Decrease the mechanical properties (modulus, hardness) of mice bones, when TGF-β signaling is augmented (Dallas et al., 1995; Balooch et al., 2005; Nyman et al., 2011)
MMP-9	Regulates VEGF (bioavailability and bioactivity)	Exerts chemotactic action on osteoclasts, which affects osteoclast recruitment during bone resorption (Bergers et al., 2000; Engsig et al., 2000; Ortega et al., 2010).
MMP-14	Activates TGF-β	Helps to preserve the survival of osteoblasts and their differentiation into osteocytes (Karsdal et al., 2002).
TGF-β	Upregulates MMP-13	Promotes bone resorption associated to changes in osteoblast morphology (Karsdal et al., 2001).
BMPs	Regulates MMP-2	Obstructs tissue remodeling and regeneration in *Poecilia latipinna* (Rajaram et al., 2016).
	Regulates MMP-9	Impairs bone remodeling (e.g., augmented bone mass during early development) and chondrocyte commitment (e.g., in the mouse C3H10T1/2 stem cell line) (Kamiya et al., 2008; Choi et al., 2009; Rajaram et al., 2016).
Wnt/β-catenin	Regulates MMP-2	Affects bone development (cartilage formation, endochondral ossification, growth plate organization, chondrocyte function) (Tamamura et al., 2005).
	Upregulates MMP-9	Modulates cartilage degradation and bone resorption (Tamamura et al., 2005).
	Regulates MMP-13	Modulates cartilage vascularization (Tamamura et al., 2005; Nakashima and Tamura, 2006; Chen et al., 2008; Papathanasiou et al., 2012).
aPC	Upregulates MMP-2 activity	Suppresses cartilage and bone degradation as well as pro-inflammatory signaling in rheumathoid arthritis patients (Nguyen et al., 2000; Buisson-Legendre et al., 2004; Xue et al., 2007).
	Downregulates MMP-9 activity	Suppresses cartilage pro-inflammatory signaling as well as cartilage and bone degradation in rheumathoid arthritis patients (Xue et al., 2007; Xue et al., 2014).

*TIMP, tissue inhibitor of MMPs; MMP, matrix metalloproteinase; TGF-β, transforming growth factor; VEGF, vascular endothelial growth factor; BMP, bone morphogenic protein; aPC, activated protein C.*

#### MMPs as Sheddases

Beyond the direct degradation of extracellular matrix substrates (e.g., collagen), MMP-mediated cleavage of substrates can lead to the release (shedding) into the extracellular matrix of soluble fragments of cell membrane-anchored receptor ligands. This extracellular event enables ligand-mediated activation of cognate receptors and elicits downstream intracellular signal transduction cascades which modify gene transcription and, ultimately, cell behavior. A prominent example pertinent to osteoblasts is the release of RANKL, which is the ligand of receptor activator of nuclear factor kappa B (RANK), by MMP-14. This MMP-14/RANKL/RANK/signal transduction axis regulates osteoblastogenesis and osteoclastogenesis, making MMP-14 crucial for normal bone formation (Bonfil et al., 2007; Thiolloy et al., 2009; Sabbota et al., 2010; Bonfil and Cher, 2011). The ligand shedding activity of MMPs influences the propensity to cancer metastasis and bone disease. For instance, MMP-14-mediated shedding of RANKL and downstream activation of RANK in the left supraclavicular lymph node cells of the prostate stimulates the non-receptor tyrosine kinase, SRC, to effectively increase the migration of prostate tumor cells which can metastasize to bone (Sabbota et al., 2010). Similarly, osteoclast-derived MMP-7 solubilizes osteoblast-bound RANKL whose release into the tumor-bone microenvironment promotes osteoclast activation in bone metastatic sites contributing to prostate and mammary tumor-induced osteolysis (Lynch et al., 2005; Thiolloy et al., 2009).

#### MMP-Generated Neoepitopes

The proteolytic action of MMPs on extracellular matrix macromolecules can result in the exposure of neo-epitopes (i.e., unique bioactive MMP-generated fragments). Compared to healthy subject controls, patients with ankylosing spondylitis (which is a form of arthritis that causes inflammation of the vertebrae) show significantly higher levels of different neo-epitopes such as C1M, C2M, C3M, C4M, C5M, C6M, and C7M from collagen type I, II, III, IV, V, VI, and VII (Veidal et al., 2012; Genovese and Karsdal, 2016). Some of these neo-epitopes have been combined (e.g., C2M, C3M, and C6M) for diagnostic purposes (Bay-Jensen et al., 2012). IPEN341-342FFGV is an MMP cleavage site which could be useful as diagnostic and prognostic makers for osteoarthritis (Bay-Jensen et al., 2011). Similarly, other MMP-generated neo-epitopes derived from collagen type II (e.g., C2C, C2M, C-terminal telopeptide of type II collagen (CTX-II), and TIINE) hold biomarker potential for osteoarthritis (Karsdal et al., 2010; Qvist et al., 2010; Karsdal et al., 2011).

#### Over-Overexpression of MMPs

Over-expression of MMPs is frequently reported in arthritis (Burrage et al., 2006; Tokito and Jougasaki, 2016). Collagenolytic MMPs (such as MMP-1, -2, -8, -13, and -14) are expressed in the arthritic joint and likely participate in the degradation of cartilage type II collagen, while MMP-3, -7, and -9 can degrade aggrecan leading to joint destruction (Puliti et al., 2012; Tokito and Jougasaki, 2016). Such a pathological mechanism has been proposed for MMP-3 and MMP-13 in degenerative joint disease in the elderly (Neuhold et al., 2001; Troeberg and Nagase, 2012; Jackson et al., 2014; Pap and Korb-Pap, 2015). Other contributions to osteoarthritis from activities related to MMP-3 include MMP-3-mediated activation of MMP-1 and MMP-13 (Mancini and di Battista, 2006; Tokito and Jougasaki, 2016). In rheumatoid arthritis, MMP-14 is greatly expressed in fibroblast-like synoviocytes and macrophages, and it could be an effector to cartilage destruction (Pap et al., 2000; Sabeh et al., 2010). MMP-1 and MMP-3 likely participate in cartilage destruction in rheumatoid arthritis and osteoarthritis (Burrage et al., 2006; Fiedorczyk et al., 2006; Tokito and Jougasaki, 2016). As a result, MMP overexpression could be therapeutically targeted in arthritis (Tokito and Jougasaki, 2016). Whether reducing MMP expression (or activity) levels provides a clinical benefit is unclear. In experimental models, many synthetic MMP inhibitors have shown positive effects (Ishikawa et al., 2005). At the clinical level, however, all efforts with MMP inhibitors to block the damaging activity of MMPs in arthritis and other non-neoplastic conditions were regrettably unsuccessful (Burrage et al., 2006; Tokito and Jougasaki, 2016). Reasons for these failures include: (i) deficient clinical trial designs (Burrage et al., 2006), (ii) unwanted characteristics of MMP inhibitors (side effects including musculoskeletal pain, low oral bioavailability, short *in vivo* half-lives, and lack of selectivity [Iyer et al., 2012; Fields, 2015; Tokito and Jougasaki, 2016]), (iii) inability of MMP inhibitors to infiltrate the cartilage/bone/synovial interface (Burrage et al., 2006), (iv) neglect of the highly complex functions served by MMPs in physiological and disease states (Iyer et al., 2012; Li et al., 2013; Sawicki, 2013) and (v) broad tissue distribution and substrate promiscuity exhibited by MMPs and their substrates (Burrage et al., 2006; Tokito and Jougasaki, 2016). To date, there remains a need for highly selective MMP inhibitors and for better information on the disease-specific substrates, which could be therapeutically targeted as shown by recent studies with MMP-13 in osteoarthritis (Li et al., 2011) as well as for more efficient and reliable techniques to sensitively measure condition-specific MMP activity potential (not just MMP expression levels).

### MMP Gene Polymorphism

A nucleotide polymorphism, by which an additional guanine creates an ETS transcription factor binding site (5′-GGA-3′) at position 1607 in the promoter sequence of the MMP-1 gene, has been related to bone mineral density (BMD) (Rutter et al., 1998). This polymorphism is associated with increased transcription of the MMP-1 gene and elevated MMP-1 activity. Among 819 postmenopausal Japanese women, BMD (e.g., D50, D100) for the distal radius had a lower value in women with the *GG/GG* genotype (47.9%) than in those with other (e.g., *G/GG* [41.9%], *G/G* [10.3%], *G/G* + *G/GG* [52.1%]) genotypes. A -1562C3 thymine polymorphism in the MMP-9 gene has been related to BMD in a population-based study (1114 Japanese men and 1087 women). It seems that the *T* allele (e.g., in men with *CT* or *TT* genotypes) of MMP-9, which shows greater transcriptional activity than the *C* allele (e.g., in men with *CC* genotype), is linked to decreased bone mass, and has a predominant effect on BMD (Zhang et al., 1999; Yamada et al., 2004). A single nucleotide polymorphism rs17576 may be involved in the pathogenesis of lumbar disk herniation (Jing et al., 2018); while the G allele of rs17576 appears to correlate with more severe stages of disk degeneration.

### MMP Deficiency and Insufficiency in Humans

Having discussed the roles of MMPs under physiological and pathological conditions, we will next discuss how their deficiency and insufficiency relates to bone metabolic abnormalities.

MMP-2 gene deficiency leads to a rare human skeletal disorder^1^, which was first reported in consanguineous Saudi Arabian families, and is characterized by severe bone alterations (Martignetti et al., 2001). Osteolytic and metabolic changes linked to MMP-2 deficiency affect tarsal, carpal, and phalangeal bones, cause severe arthropathy, osteoporosis, fibrous nodules, distinctive craniofacial defects such as exophthalmos, brachycephaly, and flattened nasal bridges and dwarfism (Al-Aqeel et al., 2000; Al-Mayouf et al., 2000; Al-Aqeel, 2005; Mosig et al., 2007; Page-McCaw et al., 2007; Castberg et al., 2013). This complex syndrome is currently categorized as a form of Torg syndrome and results from homoallelic mutations in the gene for MMP-2 located at 16q12-21 (Martignetti et al., 2001; Liang et al., 2016). A Tyr codon in the MMP-2 prodomain is replaced with the Y244X stop codon and an Arg is replaced with a His (R101H) in the cysteine-containing domain (PRCGNPD substituted by PHCGNPD). The R101H mutation is suggested to perturb coordination of Cys102 to the catalytic Zn(II) domain, consequently activating intracellular pro-MMP-2 and leading to its auto-degradation (Kennedy et al., 2005; Krane and Inada, 2008). A homoallelic missense mutation in the catalytic Zn(II) domain (E404K) has been revealed in Winchester syndrome (another variant of multicentric osteolysis) (Zankl et al., 2005). These rare Torg and Winchester arthritic syndromes together with others (such as multicentric osteolysis with nodulosis and arthropathy [known as MONA]) belong to a general family of hereditary autosomal dominant and recessive skeletal disorders with progressive bone loss and joint destruction (Al-Mayouf et al., 2000; Martignetti et al., 2001; Al-Aqeel, 2005; Zankl et al., 2005; Rouzier et al., 2006; Mosig et al., 2007; Tuysuz et al., 2009).

Similar to MMP-2, a homozygous dominant mutation (Ser substituted by Phe [F56S]) in the pro-region domain of MMP-13 also results in a bone development disorder known as spondyloepimetaphyseal dysplasia-Missouri type (Kennedy et al., 2005)^2^. This disorder, which appears to spontaneously resolve by adolescence, is characterized by anomalous modeling of long bones, mild defects in epiphysis, moderate to severe changes in the metaphysis morphology, pear-shaped vertebrae, femoral and tibial bowing, genu varum deformities, and osteoarthritis. While the biochemical mechanisms linking MMP-13 to these bone abnormalities remain unclear, the phenotype of MMP-13 deficiency could be due to a late exit of chondrocyte cells from the growth plate (Kennedy et al., 2005).

MMP-14 is widely considered one of the physiological activators of MMP-2 as it converts pro-MMP-2 into mature MMP-2 at the cell surface (Fernandez-Patron et al., 2016). An MMP-14 homoallelic mutation (T > R replacement in the signal peptide domain) destabilizes the interaction (e.g., recognition and binding) of the MMP-14 signal peptide with the signal recognition particle complex, thus affecting MMP-14 targeting to the plasma membrane (Evans et al., 2012). This MMP-14 homoallelic mutation causes an apparent deficiency of biochemically active MMP-14 at the cell membrane which impairs pro-MMP-2 activation and causes a condition of MMP-2 activity deficiency with Winchester syndrome (Evans et al., 2012)^3^.

A missense homozygous mutation (g.16250T > A, which replaces His226 of the Zn(II) catalytic domain with Gln [p.H226Q]), in the *MMP20* gene disrupts the metal-binding site and prevents MMP-20 proteolytic activity regarding enamel matrix proteins (Ozdemir et al., 2005)^4^. This mutation may lead to autosomal-recessive hypomaturation amelogenesis imperfecta, a group of inherited heterogeneous diseases that alter enamel development (amount, composition, structure) in humans (Kim J.W. et al., 2005). Another mutation in the intron 6 splice acceptor (g.30561A > T) that causes this disease is specifically characterized by pigmented teeth with a mottled and rough surface (Kim J.W. et al., 2005).

Partial loss of MMP activity or impaired MMP secretion can lead to MMP activity insufficiency. A pervasive cause of MMP insufficiency can be medications with such MMP inhibitory actions including: (i) Statins (200 million prescriptions in the United States/year; 14 million prescriptions for lovastatin alone in 2014)^5^ which can cause myositis and rhabdomyolysis (Luan et al., 2003; Thompson et al., 2003). (ii) Doxycycline (7 million prescriptions in 2014)^5^ with side-effects including joint inflammation in humans and cardiac inflammation in mice (Berry et al., 2015). (iii) Therapeutic antibodies against MMPs and MMP inhibitor drugs for treating patients with rheumatoid arthritis, severely active Crohn’s disease, and cystic fibrosis^6^. If these antibodies reduce MMP activity below baseline levels, they would cause MMP insufficiency with unpredictable consequences. Pharmacological MMP-inhibitors in Phase 3 clinical trials conducted during 1997 and 1998 in patients with advanced cancers led to an as of yet poorly understood, very severe inflammatory musculoskeletal syndrome (Zucker et al., 2000; Coussens et al., 2002). Another common cause of MMP insufficiency could be the pathological elevation of endogenous MMP inhibitors (e.g., tissue inhibitors of MMPs, α-2-macroglobulin, RECK) (Mott et al., 2000; Oh et al., 2001; Nagase et al., 2006; Klein and Bischoff, 2011). In addition, there is fibrinogen, an acute phase reactant in arthritis, which our laboratory discovered recently to inhibit MMP-2 in a cohort of rheumatoid arthritis patients (Sarker et al., 2019).

## Summary

**FIGURE 2:**
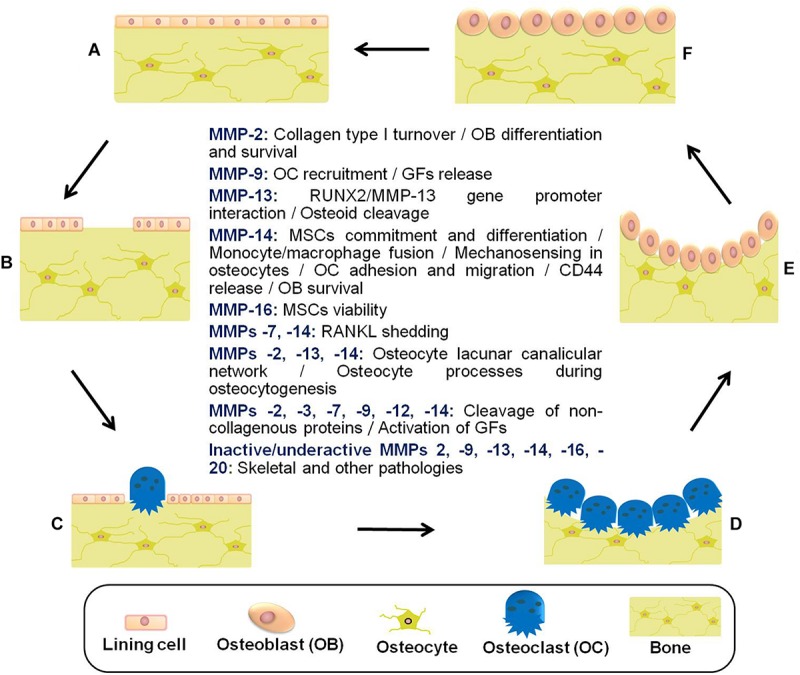
Schematic representation of the bone remodeling cycle with emphasis on the manifold roles played by matrix metalloproteinases. **(A)** Osteocytes detect mechanical stress or respond to biochemical stimuli. **(B)** Lining cells of the endosteal bone surface retract and proteases (e.g., MMPs) remove bone underlying membrane. **(C)** Osteoclasts are attracted and fused to become activated. **(D)** The underlying bone is digested by active multinucleated osteoclasts. **(E)** Osteoblasts are recruited to the bone resorption cavity. **(F)** New osteoid is formed by osteoblasts, and then mineralized (Datta et al., 2008; Fernandez-Patron et al., 2016; Paiva and Granjeiro, 2017; Cook et al., 2018). Other pathologies related to inactive/underactive MMPs are excessive inflammation, cardiovascular disorders, and metabolic dysregulation. MMP underactivity could also result from undesired side effects of common medications with MMP inhibitory actions (e.g., statins) (Cook et al., 2018). MSCs, mesenchymal stem cells; GFs, growth factors; RUNX2, runt-related transcription factor 2; RANKL, receptor activator of NF-kappa B ligand.

In summary, bone lining cells, osteocytes, osteoclasts, reversal cells, and osteoblasts are responsible for constant bone tissue remodeling (Figure 2). The activation of this multicellular unit and the intense communication between the bone cells is tightly regulated by mechanical stimuli, apoptosis, as well as systemic and local factors such as hormones and cytokines including RANKL, CSF-M, IL-3, and IL-6. Proteases of the MMP and cysteine proteinase families converge in the modulation of bone remodeling. Whereas proteolytic activity has long been thought to be required for the degradation of bone tissue in osteoarthritis and osteoporosis, inactivating mutations in MMP genes can also lead to bone pathology including osteolysis and metabolic abnormalities such as delayed growth. Thus, there remains a need to rethink the role played by proteases in bone physiology and pathology. More specific information related to bone remodeling and presumed pathways by which proteases, in particular MMPs, contribute to bone tissue remodeling in health and disease is provided in previous excellent reviews (Kini and Nandeesh, 2012; Rauner et al., 2012; Hienz et al., 2015; Liang et al., 2016; Mittal et al., 2016; Franco et al., 2017; Paiva and Granjeiro, 2017; Tauro and Lynch, 2018; Plotkin and Bruzzaniti, 2019).

## Author Contributions

EH and CF-P worked together on the conception, design, edition, revision, and approval of review manuscript.

## Conflict of Interest

The authors declare that the research was conducted in the absence of any commercial or financial relationships that could be construed as a potential conflict of interest.

## Footnotes

1 https://omim.org/entry/120360

2 https://www.omim.org/entry/600108

3 https://www.omim.org/entry/600754

4 https://www.omim.org/entry/604629

5 http://clincalc.com/DrugStats

6 http://www.gilead.com
